# Phenomenology and Clinical Correlates of Stimulus-Bound Tics in Gilles de la Tourette Syndrome

**DOI:** 10.3389/fneur.2018.00477

**Published:** 2018-06-22

**Authors:** Piotr Janik, Lukasz Milanowski, Natalia Szejko

**Affiliations:** ^1^Department of Neurology, Medical University of Warsaw, Warsaw, Poland; ^2^Department of Neurology, Faculty of Heath Science, Medical University of Warsaw, Warsaw, Poland; ^3^Department of Bioethics, Medical University of Warsaw, Warsaw, Poland

**Keywords:** stimulus-bound tics, GTS, stimulus-induced behaviors, palilalia, SBTs

## Abstract

**Introduction:** Stimulus-bound tics (SBTs) belong to stimulus-induced behaviors and are defined as tics that occur in response to internal or external stimuli. The aim of the study was to assess the prevalence and associations of SBTs with other stimulus-triggered behaviors, premonitory urges and stimulus sensitization in Gilles de la Tourette syndrome (GTS).

**Methods:** We performed a prospective, one-registration study in a cohort of 140 consecutive patients with GTS. Duration of GTS was 10.6 ± 8.7 years (range: 0–39 years). SBTs were diagnosed during the interview.

**Results:** SBTs occurred at some point in the lifetime of 20.7% of patients. The presence of SBTs in adults was four times as frequent as in children (35.5% vs. 9.0%) with the most frequent onset in adolescence (58.8%) and adulthood (29.4%). These tics started 9.1 ± 4.7 years after the onset of tics. One stimulus and mental stimulus preceded tics most frequently, 44.8 and 33.3%, respectively. There was no established pattern of tics triggered by stimuli. Multivariate logistic regression analysis showed significant associations of SBTs with age at evaluation, tic severity, and palilalia but not with any co-morbid psychiatric disorders. 80% of patients showed at least one stimulus-triggered behavior. Premonitory urges and stimulus sensitization were reported by 60.0 and 40.7% of patients, respectively. No significant correlations between SBTs, premonitory urges and stimulus sensitization were found.

**Conclusion:** SBTs are a part of the tic spectrum and should be taken into account by clinicians who deal with GTS patients. These tics fall at the tic end of the continuum of stimulus-induced behaviors.

## Introduction

Many patients with Gilles de la Tourette syndrome (GTS) are easily distracted by external stimuli. Some individuals may mirror the behavior (echopraxia) and speech (echolalia) of others as well as themselves (palilalia): they do and say what they have just seen or heard. These symptoms are called stimulus-dependent tics. To other stimulus-induced behaviors belong disinhibition behaviors (e.g., an urge to put his/her hand into the fire), stimulus sensitization (e.g., inability to wear shirts with collars because of rubbing at the neck), compulsive behaviors which belong to obsessive-compulsive disorder (OCD), or exaggerated startle responses that occur in response to both external and internal (specific thought arising in the mind, tightness in a part of the body) stimuli (1, 2). It is suggested that there is a continuum of stimulus-induced behaviors and a continuum between external and internal stimuli that induce these behaviors. However, due to lack of strict definition, the particular motor response may be interpreted in different way. For example, the urge to make a loud vocal tic in a quiet library immediately upon seeing the sign “Quiet Please” could be defined as a tic or a disinhibition behavior. Thus, we clearly defined the different stimulus-induced behaviors to separate tics triggered by stimuli from the other related behaviors. Previously, the following terms were used to describe stimulus-induced tics: reflex tics (3, 4), reflexive tics (5–8), stimulus-dependent tics (9, 10), stimulus-bound tics (SBTs) (11) or startle-induced tics (12). We decided to use the term SBTs following Leckman et al. (12) as, in our opinion, this reflects most precisely the relationship of tics and stimuli and avoids confusion with exaggerated startle response, reflex myoclonus, or other stimulus-induced behaviors. Although SBTs have been recognized as part of GTS symptomatology, little is known about how often and at which age they appear in affected individuals, what are the most common triggers that could elicit these tics, if their occurrence is related to current tic repertoire and severity, other stimulus-induced behaviors (premonitory urges and stimulus sensitization) or co-morbid psychiatric disorders. The study aimed to answer all questions above and fulfil the gap in knowledge on SBTs in GTS.

## Materials and methods

### Study participants

The cohort of GTS cases comprised 140 consecutive patients aged 5–50 years (mean age: 17.9 ± 10.0 years; 107 males, 76.4%). The subjects were evaluated from 2013 to 2017. Seventy-eight children (55.7%, mean age: 10.4 ± 3.1 years), and 62 adults (mean age: 27.2 ± 7.4 years) were enrolled. The mean age of tic onset was 6.5 ± 2.8 years. Duration of GTS was 4.9 ± 3.0 years (range: 0–13) in children and 18.7 ± 7.5 years (range: 6–39) in adults. One hundred and sixteen (82.9%) patients had, at least, one psychiatric co-morbidity (Table 1). The patients were evaluated for the clinical diagnosis of GTS and co-morbid mental disorders according to DSM-IV-TR. OCB was diagnosed if obsessions and compulsions were egosyntonic in contrast to egodystonic symptoms which characterized OCD. The diagnosis of co-morbid mental disorders was also made based on earlier psychiatric examinations that had been performed before the time of patients' evaluation. This included psychiatric disorders that were usually diagnosed in the childhood (e.g., attention deficit hyperactivity disorder or oppositional defiant disorder) and which symptoms were not yet present in adult patients at the time of examination. However, it applied only to few subjects. All the patients were referred to neurologists experienced in movement disorders and were personally interviewed by the author of the study (PJ). The study was designed as a one-time registration study and no new clinical data obtained on follow-up visits were included in the analysis.

**Table 1:** Characteristics and associations of SBTs.

	**GTS patients (*****n*** = **140)**		
	**Patients with SBTs(*n* = 29)**	**Patients without SBTs (*n* = 111)**	***p***
Age at evaluation [years] (median, IQR)	25 (18–32)	12 (9–22)	0.00005
YGTSS [median] (IQR)	64 (58–79)	40 (24–54)	0.00001
Palilalia	48.3% (*n* = 14)	13.5% (*n* = 15)	0.00014
Coprolalia	51.7% (*n* = 15)	17.1% (*n* = 19)	0.0004
Copropraxia	17.2% (*n* = 5)	5.5% (*n* = 6)	0.052
Echolalia	44.8% (*n* = 13)	11.7% (*n* = 13)	0.0002
Echopraxia	20.7% (*n* = 6)	6.4% (*n* = 7)	0.03
Total number of complex tics [median] (IQR)	10 (4.5–13)	6 (3–10)	0.04
Depression	34.5% (*n* = 10)	11.7% (*n* = 13)	0.009
Attention-Deficit Hyperactivity Disorder	17.2% (*n* = 5)	28.8% (*n* = 32)	0.24
OCD/OCS	65.5% (*n* = 19)	58.6% (*n* = 65)	0.53
Non-OCD Anxiety Disorder	51.7% (*n* = 15)	49.6% (*n* = 55)	1.00
Learning Disorder	55.2% (*n* = 16)	60.4% (*n* = 67)	0.67
Conduct Disorder/ Oppositional Defiant Disorder	10.3% (*n* = 3)	9.9% (*n* = 11)	1.00
Aggression	20.7% (*n* = 6)	36.9% (*n* = 41)	0.12
Self-injurious behavior	31.0% (*n* = 9)	36.0% (*n* = 40)	0.67
Significant social skills problems	44.8% (*n* = 13)	51.4% (*n* = 57)	0.68
Premonitory urges	72.4% (*n* = 21)	57.8% (*n* = 63)	0.2
Stimulus sensitization	35.7% (*n* = 10)	43.1% (*n* = 47)	0.53
Family history of tics or GTS	65.5% (*n* = 19)	65.5% (*n* = 72)	1.00
Family history of OCD or OCS	17.2% (*n* = 5)	31.8% (*n* = 35)	0.17
Medication for tics [at evaluation]	48.3% (*n* = 14)	39.6% (*n* = 44)	0.41

*n,number of patients; IQR,interquartile range; p,SBTs+ vs. SBTs-. SBTs,stimulus bound tics; YGTSS,Yale Global Tic Severity Scale; OCD, obsessive-compulsive disorder; OCS, obsessive-compulsive symptoms*.

### Definition and differential diagnosis of SBTs

During the interview, patients were asked if they have had tics, or had in the past, in response to something they have just seen, heard or thought. They were questioned about kind of tics following a stimulus, whether or not they would occur spontaneously (without preceding stimulus), and at which age they started. SBTs were defined as a tic or tics triggered by a specific stimulus while the type of stimulus and the tic had to be different. Therefore, subjects who experienced echolalia, echopraxia, and palilalia were excluded from SBTs+ group, and, as patients having more typical tics, they were included into SBTs- group. Diagnosis of SBTs in two young children was made on observation of their behavior during clinical examination. These children did not report these tics themselves.

We differentiated SBTs with regard to premonitions and motor response. Subjects were asked questions about (1) premonitory urges preceding tics, defined as an internal sensation (itch, stretch, tightness, tingling), in those parts of the body where the tics occur; (2) stimulus sensitization, defined as heightened sensitivity to tactile, auditory, and visual stimuli that resulted in uncomfortable sensation, tension, or non-tic movement; (3) compulsive behaviors, defined as urge-driven complex behavior aimed at reducing anxiety or distress; (4) disinhibition behaviors, defined as urge-driven, complex and purposeful behaviors that are dangerous, forbidden, socially inappropriate or senseless and bizarre; (5) exaggerated startle responses, defined as movements or vocalizations following unexpected and sudden stimulus. Subjects who experienced these phenomena were defined as not having SBTs and included in the SBTs-group. Compulsive behaviors were included into the OCD group. None of the patients had exaggerated startle responses during the examination.

### Statistical analysis

The statistical analyses were performed using STATISTICA ver 13.1 software. Normality of distribution was assessed using the Shapiro-Wilk test. Continuous variables are presented as median and (IQR). Categorical variables are presented as frequencies (percentages). The parametric data were compared by independent *t*-test and the nonparametric data by the Mann–Whitney *U*-test, as appropriate, and the categorical data were compared by Fisher's exact test (two-sided).

Logistic regression model were created to determine factors associated with SBTs. In first step we performed univariate analysis of SBTs with different demographic and clinical data. Selected data which reached *p*-value less than 0.05 in univariate analysis were included in the multivariate analysis to create the final model. Model discrimination was determined by calculating the area under the receiver operating characteristic curve (AUC). A value of *p* ≤ 0.05 was considered significant for all tests (13).

## Results

**Figure 1:**
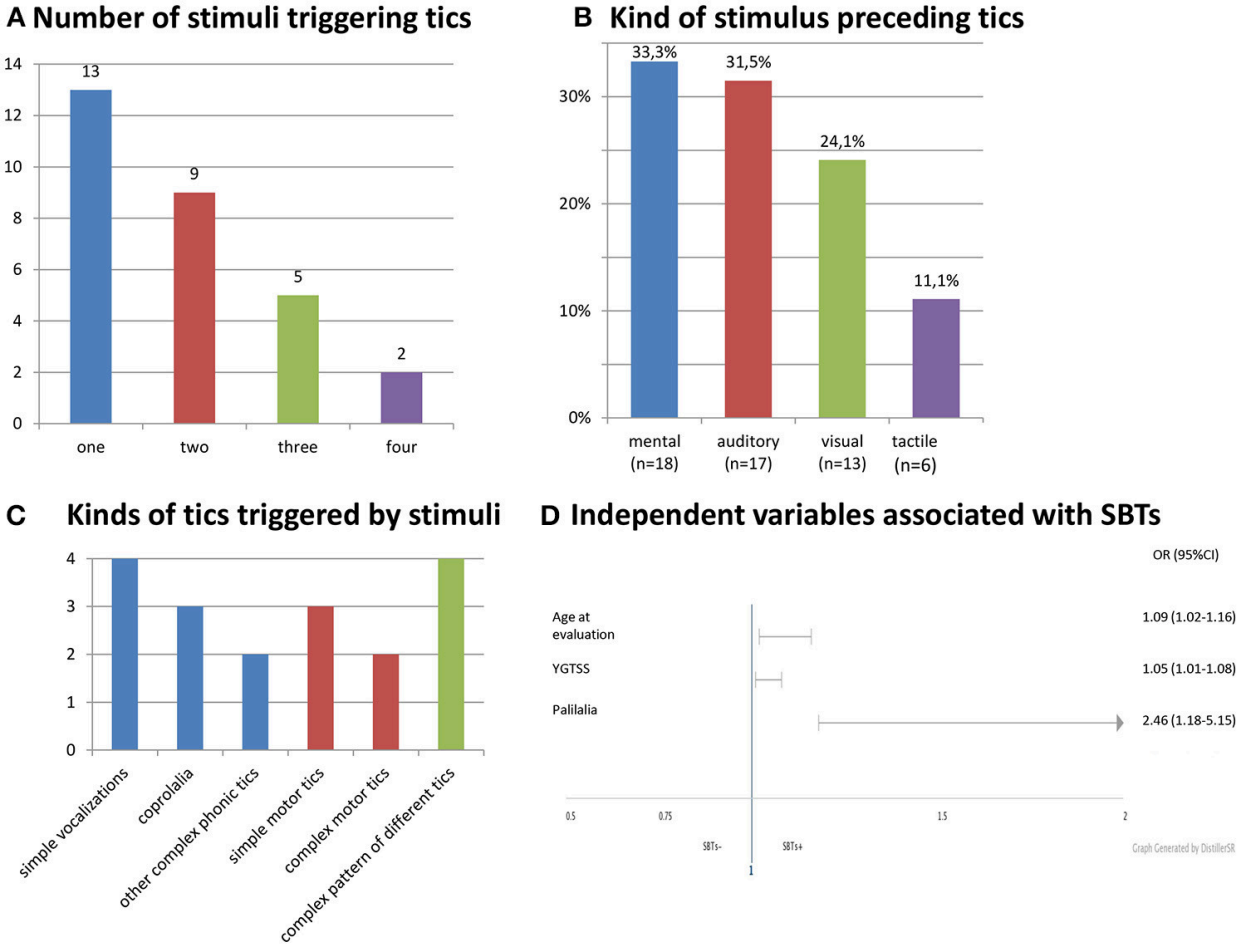
Characteristics and stimuli and SBTs. **(A)** Number of stimuli triggering tics. **(B)** Kind of stimulus preceding tics. **(C)** Kinds of tics triggered by stimuli. **(D)** Independent variables associated wit SBTs.

Stimulus-bound tics occurred in 20.7% (29/140) of all patients, 35.5% (22/62) of adults, 9.0% (7/78) of children. SBTs were reported in our group three times more often by adults (22/29, 75.9%; *p* = 0.00014) than children. The patients with SBTs were older and had more severe tics (Table 1). Gender did not differ SBTs+ and SBTs- groups (males, 65.5 vs. 79.3%; *p* = 0.14). SBTs were continuing in 28 patients at the time of evaluation. In only one patient, SBTs disappeared entirely before the clinical evaluation. Age at onset of SBTs was known in 17 patients (mean: 16.0 ± 5.5 years; range: 7–26). SBTs started in two children (7–11), 10 adolescents (12–18), and five adults (≥18 years). Age of SBTs onset was 9.1 ± 4.7 years (range: 1–20) after tic onset. In one patient only, SBTs appeared in the first 3 years of the disease. Characteristics of stimuli and SBTs are shown in Figure 1.

In univariate logistic regression, SBTs were significantly correlated with an older age at the time of evaluation, more severe and complex tics, presence of coprophenomena, echophenomena, and palilalia. Among psychiatric co-morbidities, only depression was associated with SBTs. OCD/OCB, premonitory urges and stimulus sensitization were not related to SBTs. In multivariate logistic regression, only age at the time of examination, YGTSS score, and palilalia remained significant (Table 2). The AUC for this model was 0.8820 and reached a value >0.7, which is the minimal value to consider the model fairly discriminated.

**Table 2:** Logistic regression model for SBTs occurence.

	**Univariate analysis**		**Multivariate analysis**	
	**OR (95% CI)**	***p***	**OR (95% CI)**	***p***
Age at evaluation	1.09 (1.05–1.14)	0.00005	1.09 (1.02–1.16)	0.015
YGTSS	1.06 (1.03–1.09)	0.000005	1.05 (1.01–1.08)	0.010
Palilalia	2.44 (1.55–3.85)	0.0001	2.46 (1.18–5.15)	0.017
Coprolalia	2.28 (1.47–3.54)	0.00025	1.62 (0.81–3.22)	0.172
Copropraxia	1.90 (1.01–3.58)	0.047	1.45 (0.58–3.60)	0.428
Echolalia	2.47 (1.55–3.95)	0.00014	1.39 (0.68–2.83)	0.364
Echopraxia	1.96 (1.09–3.54)	0.0255	1.59 (0.70–3.60)	0.269
Total number of complex tics	1.09 (1.00–1.19)	0.047	0.90 (0.76–1.06)	0.203
Depression	1.99 (1.23–3.22)	0.0049	0.78 (0.36–1.71)	0.539

*SBTs, stimulus bound tics; YGTSS-Yale Global Tic Severity Scale*.

We also analyzed all sensory-triggered behaviors and found that 80.0% (112/140) of GTS patients had at least one of them. The patients who did not report any of these behaviors (28/140) were younger at evaluation (mean: 13.3 ± 8.9 vs. 19.0 ± 9.9 years, *p* = 0.0016) and had shorter disease duration (mean: 6.4 ± 8.9 vs. 11.6 ± 8.3 years, *p* = 0.0002). Tic severity measured by YGTSS and co-morbid psychiatric disorder rates did not differ both groups. Premonitory urges and stimulus sensitization were reported by 60% (84/140) and 40.7% (57/140) of patients, respectively. Combination of SBTs and premonitory urges were seen in 15% (21/140), SBTs and stimulus sensitization in 7.1% (10/140), and stimulus sensitization and premonitory urges in 23.6% (33/140) of patients. We did not find any significant associations of SBTs with premonitory urges (*p* = 0.2) and stimulus sensitization (*p* = 0.53). There were also no correlations between premonitory urges and stimulus sensitization (*p* = 0.21). We did not record disinhibition behavior although there were only few patients with such a problem in our cohort.

The raw data underlying the findings of the study are presented in S1 Table as Supplementary Material.

## Discussion

Stimulus-bound tics occurred at some point in the lifetime of 1/5 of the patients. The incidence of SBTs was strongly dependent on patients' age. More than 1/3 of adults and only one of ten children had these tics which means that SBTs were four times more frequent in adults than in children. It remains unclear whether those tics really are a late symptom of GTS or children are simply not aware of them. During the interview, young children had difficulty understanding questions about tics occurring in response to different stimuli. This may explain why SBTs had not been thoroughly examined because most studies in GTS addressed child population. In our cohort adult patients represented nearly half of all GTS patients. The onset of SBTs was mainly in adolescence but they may appear life-long or even after 20 years of the duration of the disease.

Stimulus-bound tics seem to be fully ego-syntonic. They were never reported by the patients themselves and were realized only due to the active inquiry of a physician, and many patients (12/29) were not able to give the exact age of onset. From the positive correlation of SBTs with YGTSS score, we can speculate that severe tics may be associated with SBT risk or SBTs could substantially contribute to tic severity. Thus, it is possible that SBTs may add significantly to the impairment caused by tics. However, except two subjects, SBTs were never reported as the most troublesome symptom of the disease and were not related to more frequent use of medication for tics (Table 1). We also suspect that SBTs had been unrecognized and that's why the patients with this kind of tics were not treated more intensively.

We separated echophenomena and palilalia from SBTs because tic response and stimulus preceding tics are of the same nature, and we tested the hypothesis if these tics may comprise the same tic spectrum. Palilalia means that people with GTS mirror the speech of themselves in response to their own auditory stimulus. In our study, SBTs were significantly associated with palilalia (Table 2), so we suppose that these tics may belong to the same tic spectrum. Even though the association of echophenomena, coprophenomena, and complex tics with SBTs was not confirmed in multivariate analysis, we suggest that echo- and coprophenomena may be within the continuum of SBTs, and SBTs may belong to complex tics (Table 2).

We did not find any specific pattern of SBTs. The patients developed one or many different tics (Figure 1C). They might appear spontaneously (without stimulus) or only in response to a stimulus. SBTs were elicited by both external and internal stimuli. A mental stimulus such as specific thought or discrete reminiscence of a particular event was the most common trigger (Figure 1B). Premonitory urges and distress preceding compulsions that were found to be related to OCD encompass other internally arising stimuli (14, 15). However, we did not find an association of SBTs with premonitory urges, OCD/OCB or any co-morbid psychiatric disorders. There was also no correlation between SBTs and significant social skill problems or stimulus sensitization which are a part of the autistic spectrum disorder phenomenology. That is why we think that SBTs fall at the tic end of the continuum of stimulus-induced behaviors.

If we analyzed all studied sensory-triggered behavior we found that 4/5 of patients had at least one of them. We were able to diagnose these behaviors probably because young children from whom it is difficult to receive reliable information represented minority of our patient group. What is not surprising, premonitory urges were most frequently reported. SBTs were diagnosed twice less often compared to stimulus sensitization. We cannot confirm that all studied stimulus-induced behaviors belong to the same clinical spectrum because we did not find any significant correlations between premonitory urges, stimulus sensitization, and SBTs. The reason of this may be a small number of individuals with each sensory-triggered behavior.

Our study focuses on sensations preceding tics that are often not reported spontaneously and that could be easily overlooked. On the other hand, they can cause significant deterioration of patient's quality of life. Further studies including larger study sample are needed to elucidate if these behaviors are actually as frequent as in our group and underlying biological processes that lead them to appear are common or not.

Limitations include different methodology of collecting data from children and adults. Most clinical information regarding children are provided by their parents whereas adults report themselves. Data obtained from adults were retrospective with the possibility of recall bias. The data regarding clinical correlates of SBTs should be interpreted with caution due to small number of cases in stimulus-induced behavior and co-morbid psychiatric disorder groups. One-time registration study design may have influenced the prevalence of SBTs. There is also possible referral bias because the patients were evaluated by neurologist and the cases with more severe psychopathology were referred to psychiatric clinics.

We conclude that SBTs, echophenomena and coprophenomena may be a part of the same complex tic spectrum. Although they appear in minority of GTS patients, they may add significantly to tic severity and that's why should be sought by active inquiry during the interview. SBTs are likely to be unrecognized and therefore it does not translate to treatment intensification. Other sensory premonitions like premonitory urges and stimulus sensitization were found to occur in most patients with GTS. Further studies are needed to elucidate if these sensations are related to the impairment caused by GTS.

## Ethics statement

Data regarding SBTs and phenomenology of GTS were obtained during standard clinical examination. Collecting of clinical data from patients with GTS has been approved by the Ethics Committee of Medical University of Warsaw (KB/2/2007). Each patient gave written informed consent to use the data for scientific research.

## Author contributions

PJ: Conceived and designed of the study. PJ: Acquired data. NS and LM: Set up the electronic database. LM and PJ; Analyzed and interpreted the data. LM: Performed visualization: PJ: Wrote the original draft of the manuscript. LM, NS, and PJ: Reviewed and edited the manuscript.

### Conflict of interest statement

The authors declare that the research was conducted in the absence of any commercial or financial relationships that could be construed as a potential conflict of interest.

## Supplementary material

S1 Table The raw data underlying the findings of the study.
