# Supplementary material for: Phenomenology and Clinical Correlates of Stimulus-Bound Tics in Gilles de la Tourette Syndrome
Source: Front Neurol. 2018 Jun 22;9:477. doi: 10.3389/fneur.2018.00477 (PMC6024546; doi:10.3389/fneur.2018.00477)
Supplement: S1 Table — The raw data underlying the findings of the study. [file Data_Sheet_1.PDF]

Consecutive patient	Stimulus bounds tics 1-yes 0-no	Kind of stimulus 1-visual; 2-auditory; 3-tactile; 4-mental	Age at evaluation (yrs)	Age at onset of SBTs	Disease duration (years)	Gender 0-male 1-female	total number of complex tics
1	0		38			0	1
2	0		22			0	11
3	1	1;2;3	30		23	0	
4	1	2;3	27		20	0	2
5	1	4	32		26	0	10
6	0		20		9	0	5
7	0		27		21	0	8
8	0		37		32	0	5
9	1	1;2;4	21	17	13	0	12
10	0		22		10	0	7
11	0		23		21	0	10
12	1	2;4	25	23	12	0	8
13	0		25		14	1	2
14	0		22		12	0	6
15	1	4	31			1	5
16	0		19		11	0	2
17	0		21		6	0	12
18	0		35		20	0	6
19	0		38			0	0
20	0		32		27	0	17
21	0		27		23	0	0
22	1	4	18	16	12	0	11
23	0		27		19	0	1
24	0		23		19	0	14
25	0		17		12	0	7
26	0		21		14	0	1
27	1	2;4	29		23	1	5
28	0		17		3	0	2
29	0		23		18	0	9
30	0		35		25	1	4
31	1	1	25	25	18	1	13
32	0		32			1	2
33	1	3	25		20	0	2
34	0		22		17	1	10
35	0		22		12	1	0
36	0		21		15	0	13
37	1	3;4	38		31	1	4
38	0		18		8	0	10
39	0		18		6	1	4
40	0		25		16	0	8
41	0		37		23	0	16
42	1	1;2;4	21	16	13	0	14
43	1	4	40	26	34	1	2
44	1	4	35	19	27	0	13

45	0		44		39	0	3
46	0		26		12	0	6
47	0		33		26	0	1
48	1	2;4	33		26	0	10
49	0		26		20	0	1
50	1	1;2;3;4	18	12	10	1	14
51	1	4	50			0	6
52	1	1;2;4	33		21	1	14
53	0		18		16	0	7
54	0		22		12	1	4
55	0		20		13	0	0
56	0		6		1	0	8
57	0		11		1	1	2
58	0		9		2	1	8
59	0		10		6	0	6
60	1	2	16	16	12	0	13
61	0		5		0	0	14
62	0		10		4	1	10
63	0		7		3	1	17
64	0		7		2	0	3
65	0		5		2	0	2
66	0		12		8	0	13
67	0		6		1	1	7
68	0		16		11	0	12
69	0		5		2	0	7
70	0		10		3	0	6
71	0		8		1	0	11
72	0		8		5	0	23
73	0		7		1	0	11
74	0		11		5	0	4
75	1	1;2;3;4	18		12	0	15
76	0		10		7	0	4
77	0		12		2	1	5
78	0		9		5	0	7
79	0		7		0	0	17
80	0		8		4	1	3
81	0		12		7	0	8
82	0		8		4	1	2
83	0		10		1	0	9
84	0		9		4	0	14
85	0		11		6	1	9
86	0		8		5	0	2
87	1	2	8	7	2	0	11
88	0		15		8	0	9
89	0		13		10	0	4
90	0		8		2	0	14
91	0		12		2	0	3
92	0		10		4	0	1
93	0		15		9	0	10
94	0		15		6	0	5
95	0		17		9	1	8
96	0		10		6	0	0
97	1	1;2	12	12	4	1	2

98	0		10		5	1	7
99	0		9		3	0	11
100	0		15		3	0	16
101	0		9		4	0	6
102	0		11		9	0	13
103	0		8		2	0	6
104	0		8		4	0	2
105	1	1	14	12	10	0	17
106	0		12		7	0	10
107	0		8		4	0	7
108	0		10		7	0	11
109	1	1;2	13	12	7	0	
110	0		14		7	0	13
111	0		8		2	0	4
112	0		8		5	0	9
113	0		13		5	1	0
114	0		12		3	0	10
115	0		11		6	0	7
116	0		9		4	0	10
117	0		9		2	0	9
118	1	4	14	13	10	0	15
119	0		7		2	1	6
120	0		29		22	0	4
121	1	1;4	41		34	0	13
122	0		19		12	1	3
123	0		9		4	1	6
124	0		25		22	0	3
125	0		11		6	0	5
126	0		8		2	0	3
127	0		20			1	4
128	1	2	23	22	11	1	3
129	1	1;2;4	31	15	25	0	3
130	0		11		7	0	12
131	0		6		1	0	0
132	0		11		4	0	3
133	0		10		6	0	5
134	0		15		7	0	10
135	0		16		13	0	2
136	0		29		24	0	18
137	0		10		6	0	7
138	0		9		5	0	6
139	1	1;2	9	9	6	1	6
140	0		14		9	0	4

S1 Table. Dataset of raw values underlying the findings of

YGTSS-TTS	YGTSS-Impairment	YGTSS total	coprolalia 1-yes 0-no	copropraxia 1-yes 0-no	echolalia 1-yes 0-no	echopraxia 1-yes 0-no	palilalia 1-yes 0-no	depression 1-yes 0-no
25	10	35	0	0	0	0	0	0
22	20	42	0	0	0	0	1	0
47	40	87	1	0	1	0	1	1
25	10	35	1	0	0	0	0	1
32	30	62	0	0	0	0	1	1
36	40	76	0	0	0	0	0	1
21	0	21	0	0	0	0	0	0
31	40	71	0	0	0	0	0	1
30	30	60	0	0	0	0	1	0
23	20	43	0	0	1	1	0	0
33	40	73	0	0	0	0	0	1
35	30	65	0	0	1	0	1	0
24	20	44	0	0	0	0	0	0
28	30	58	0	0	0	0	0	0
31	40	71	0	0	0	0	1	1
28	20	48	0	0	0	0	0	0
29	20	49	0	0	0	0	0	0
26	20	46	0	0	0	0	0	0
14	0	14	0	0	0	0	0	0
22	10	32	0	0	0	0	0	0
24	10	34	0	0	0	0	0	0
40	20	60	0	0	0	0	0	0
13	10	23	0	0	0	0	0	0
33	30	63	0	0	1	0	1	1
23	20	43	0	0	0	0	0	0
22	30	52	0	0	0	0	0	0
17	10	27	0	0	1	1	1	0
17	0	17	0	0	0	0	0	0
26	30	56	0	0	0	0	0	1
25	20	45	0	0	0	0	0	0
38	40	78	1	0	1	0	1	1
16	10	26	0	0	0	0	0	0
23	10	33	0	0	0	0	0	0
22	50	72	0	0	0	0	0	1
12	20	32	0	0	0		0	0
42	50	92	1	0	0	0	0	0
36	40	76	0	0	0	0	0	1
28	30	58	0	0	0	0	0	1
19	20	39	0	0	0	0	0	0
33	20	53	0	0	1	0	0	0
41	30	71	0	0	1	0	1	1
32	30	62	1	1	0	0	0	0
28	30	58	1	0	0	0	0	1
37	50	87	1	0	1	1	1	1

40	30	70	1	0	0	0	0	0
31	30	61	0	0	0	0	1	1
20	20	40	0	0	0	0	0	0
43	50	93	1	1	1	1	0	0
13	20	33	0	0	0	0	0	1
43	40	83	1	1	1	0	1	1
25	30	55	0	0	0	0	1	0
42	30	72	1	0	0	0	1	1
38	30	68	1	0	0	1	0	0
24	20	44	0	0	0	0	0	0
17	0	17	0	1	0	0	0	0
25	10	35	0	0	0	0	0	0
15	0	15	0	0	0	0	0	0
18	0	18	1	0	0	0	0	0
19	0	19	0	0	0	0	0	0
40	40	80	0	0	1	0	0	0
34	20	54	1	0	1	0	1	0
20	0	20	0	0	0	0	1	0
27	10	37	0	0	0	0	0	0
22	20	42	0	0	0	0	0	0
9	0	9	0	0	0	0	0	0
27	30	57	0	0	1	0	0	0
28	10	38	0	0	0	0	1	0
26	20	46	0	0	0	0	0	1
19	0	19	0	0	0	0	1	0
24	0	24	1	0	0	0	0	0
24	0	24	0	0	0	0	0	0
34	20	54	1	0	1	1	1	0
20	10	30	1	0	0	0	0	0
20	20	40	0	0	0	0	0	0
49	30	79	1	0	1	0	0	0
26	10	36	0	0	0	0	0	0
27	40	67	0	0	0	0	0	0
37	30	67	0	0	0	0	0	0
27	0	27	0	0	0	0	1	0
24	0	24	0	0	0	0	0	0
23	20	43	0	0	1	0	0	0
20	0	20	0	0	0	0	0	0
22	20	42	0	0	0	0	0	0
31	0	31	0	0	0	1	0	0
14	0	14	0	0	0	0	0	0
16	10	26	0	0	0	0	0	0
27	0	27	0	0	1	1	1	0
35	30	65	0	0	0	1	0	0
17	10	27	0	0	0	0	0	0
29	30	59	1	1	1	0	1	0
23	20	43	0	0	0	0	0	0
7	20	27	0	0	0	0	0	0
22	0	22	0	0	0	0	0	0
19	10	29	0	0	0	0	0	0
18	0	18	0	0	0	0	0	1
20	20	40	0	0	0	0	0	0
15	0	15	0	0	0	0	0	0

13	0	13	1	0	0	0	0	0
33	20	53	1	0	0	0	1	0
35	30	65	1	0	0	0	0	0
18	10	28	0	0	0	0	0	0
40	30	70	1	1	0	0	0	0
30	20	50	0	0	0	0	0	0
23	10	33	0	0	0	0	0	0
34	30	64	1	1	1	0	0	0
39	40	79	1	0	1	0	0	0
20	20	40	0	0	0	0	0	0
21	20	41	0	0	0	0	1	0
39	40	79	0	0	0	0	0	0
23	10	33	0	0	0	0	0	0
17	0	17	0	0	0	0	0	0
40	40	80	1	0	1	0	0	0
17	20	37	0	0	0	0	0	0
38	30	68	0	1	0	0	0	0
24	30	54	0	0	0	0	0	0
30	30	60	0	0	0	0	0	0
28	30	58	0	0	0	0	1	0
33	30	63	1	0	0	0	1	0
31	30	61	0	0	0	0	0	0
23	10	33	0	0	0	0	0	0
42	30	72	1	0	1	0	1	0
23	20	43	0	0	0	0	0	0
15	0	15	0	0	0	0	0	0
10	10	20	0	0	0	0	0	0
23	10	33	0	0	0	0	0	0
19	0	19	0	0	0	0	0	0
21	0	21	1	0	1	0	0	0
21	40	61	1	0	0	0	0	0
31	20	51	0	0	0	1	0	0
33	20	53	0	0	0	0	0	0
17	0	17	0	0	0	0	0	0
18	20	38	0	0	0	1	0	0
8	0	8	0	0	0	0	0	0
42	20	62	1	1	0	0	0	0
18	0	18	0	0	0	0	0	0
17	0	17	1	1	1	1	1	1
17	0	17	0	0	0	0	0	0
25	10	35	1	0	0	0	0	0
46	40	86	1	1	1	1	0	0
31	20	51	0	0	0	0	0	0

: the study

Premonitory urges 1-yes 0-no	Family history of tics or GTS 1-yes 0-no	Family history of OCD or OCS 1-yes 0-no	ADHD 1-yes 0-no	OCD + OCS 1-yes 0-no	Non-OCD Anxiety Disorder 1-yes 0-no	Aggression 1-yes 0-no	Self-injurious behaviour 1-yes 0-no	Stimulus sensitization 1-yes 0-no
1	1	1	0	0	0	0	0	0
0	0	0	1	1	1	1	0	0
1	1	1	0	0	0	0	1	0
1	0	0	0	1	1	0	1	0
1	0	0	0	0	1	0	1	0
0	0	0	1	1	0	0	0	0
1	1	1	0	1	1	0	0	1
0	0	0	0	0	0	0	0	0
1	1	1	0	1	1	0	0	1
1	0	0	0	1	0	0	1	0
1	1	0	1	0	0	0	1	1
0	1	0	0	0	0	0	0	
1	1	0	0	0	0	0	0	0
1	1	1	1	1	0	0	0	1
0	0	0	0	0	0	0	0	0
1	0	0	0	1	1	0	0	1
1	0	0	0	1	1	0	0	1
1	0	0	1	1	1	1	0	1
	1	0	0	0	0	0	0	0
1	1	0	0	1	1	0	0	1
0	1	1	1	1	1	1	1	1
1	1	0	0	1	1	1	1	1
1	1	0	0	1	1	0	0	1
1	1	0	0	1	1	1	1	1
1	1	0	0	1	0	1	1	1
1	0	0	1	1	1	1	1	0
1	1	0	0	1	0	0	0	0
0	1	1	0	1	1	0	0	0
1	1	1	1	1	1	1	1	1
1	1	0	0	1	0	1	1	0
1	1	0	0	1	1	0	0	0
1	1	0	0	0	0	0	0	0
1	0	0	1	0	1	1	0	1
1	1	1	1	1	0	0	1	1
1	1	0	0	0	0	0	0	1
1	1	1	1	1	1	1	1	0
1	1	1	0	1	1	1	0	1
1	1	1	0	0	1	0	0	1
0	0	0	0	1	0	0	1	0
1	1	0	1	1	1	1	0	1
1	0	0	0	0	0	1	1	0
1	1	0	1	0	0	0	0	0
1	1	1	0	1	1	0	0	0
1	1	0	0	0	0	0	0	0

0	1	1	1	0	0	0	0	0
1	1	0	0	0	0	0	1	0
1	1	0	0	0	1	0	0	0
1	1	0	0	0	1	0	0	1
1	1	1	0	1	1	0	0	1
1	1	0	0	1	1	0	0	0
1	0	0	1	1	0	0	1	0
1	1	0	0	1	0	0	0	1
1	0	0	1	1	0	1	1	1
1	0	0	0	0	0	0	0	0
0	1	0	1	0	1	0	0	1
0	1	0	0	0	1	0	0	0
1	1	0	0	0	1	0	0	0
0	1	0	0	0	0	0	0	0
1	1	0	0	1	0	0	0	1
0	1	0	1	1	1	1	1	0
1	1	0	1	1	1	1	0	1
1	0	0	1	0	0	1	1	0
0	1	0	0	1	1	0	1	1
0	0	0	0	0	0	1	0	1
0	1	1	1	1	1	1	1	1
1	1	0	0	0	0	0	0	0
1	1	1	1	0	0	1	0	1
1	0	1	0	1	1	0	1	1
0	1	0	0	0	0	0	0	0
1	0	0	0	0	0	1	0	0
0	1	1	1	1	1	1	1	0
0	0	0	1	1	1	1	1	0
0	0	0	1	1	1	0	1	1
1	1	0	0	0	1	1	1	0
1	1	0	0	1	0	0	0	0
0	0	0	0	1	1	1	0	0
0	0	0	0	0	0	0	0	0
1	1	0	0	1	0	0	0	1
0	0	1	0	1	1	1	0	0
0	1	0	0	0	0	0	0	0
1	1	1	0	1	0	0	0	0
1	1	0	1	0	1	0	0	1
0	0	1	0	1	0	0	0	0
0	1	1	0	0	0	0	0	0
0	1	1	0	1	1	0	1	1
1	0	0	0	0	0	1	0	1
1	0	0	0	1	0	0	0	1
1	1	0	0	0	0	0	0	0
0	1	1	1	1	1	0	1	1
1	1	0	0	0	0	0	0	1
1	1	0	0	0	0	0	0	0
1	1	1	0	1	1	1	1	1
0	1	0	0	0	0	0	0	
1	0	0	0	0	0	0	0	0
1	0	0	0	0	0	0	0	0
1	1	0	0	0	0	0	0	1
0	1	0	0	1	1	1	0	1

0	1	1	0	1	0	0	0	1
0	1	1	1	1	1	1	0	1
1	1	0	0	1	0	0	0	0
0	0	1	0	0	1	0	1	1
0	1	0	0	1	1	0	0	
1	1	0	0	0	0	0	0	0
0	0	1	0	1	1	0	0	1
1	0	0	0	1	0	0	1	0
1	0	0	1	0	0	1	0	0
1	1	0	0	0	1	0	0	0
1	1	1	0	1	0	0	0	0
1	0	0	0	1	0	0	0	1
	0	1	0	1	1	0	1	0
0	0	0	0	1	1	1	1	1
0	0	0	1	1	1	1	1	1
0	0	0	1	1	1	0	1	0
0	1	0	0	1	0	1	0	0
1	1	1	0	0	0	0	0	0
1	1	0	0	1	1	0	0	0
0	1	1	0	1	0	1	0	0
0	0	0	0	1	1	0	0	0
0	1	0	0	1	0	1	0	0
1	0	1	1	1	1	1	1	0
0	1	1	1	1	1	0	1	0
0	1	0	0	1	0	1	0	0
1			1	1	1	1	1	1
1	1	1	0	1	1	0	1	0
0	1	0	0	0	0	0	0	0
0	0	0	1	1	1	1	1	1
1	0	0	0	0	1	0	1	0
1	1	0	0	0	0	0	1	0
0	1	0	0	0	0	0	0	1
0	0	0	0	1	0	0	0	0
0	1	0	1	0	1	1	1	0
0	0	0	0	0	0	0	0	1
0	1	0	0	1	0	0	0	0
0	1	1	1	1	1	0	1	0
1	1	0	0	0	0	1	0	0
1	1	0	0	1	1	1	1	1
1	1	1	0	1	1	1	1	1
0	0	0	0	1	1	0	0	0
0	0	0	0	1	1	1	0	0
1	1	1	0	1	1	1	1	1

Conduct disorder/ oppositional defiant disorder 1-yes 0-no	Significant social skill problems 1-yes 0-no	Learning disorder 1-yes 0-no	Medication for tics 1-yes 0-no
0	0	1	1
0	1	1	0
0	1	1	1
0	1	1	1
0	1	1	1
0	0	0	1
0	0	1	1
0	0	0	1
0	0	1	1
0	1	0	1
0	1	0	1
0	0	0	0
0	0	0	0
1	0	1	1
0	0	0	1
0	0	1	1
0	0	1	1
0	1	1	1
0	0	0	0
0	0	1	1
1	1	1	1
1	1	1	1
0	0	1	1
0	1	1	1
0	1	0	1
0	1	1	0
0	0	0	1
0	0	1	1
1	1	1	0
0	1	0	0
0	0	1	1
0	0	0	0
0	1	1	1
0	1	1	1
0	0	0	0
0	1	1	0
0	1	1	1
0	0	1	0
0	1	0	0
0	1	1	0
0	1	0	1
0	0	0	0
0	0	1	0
0	0	0	1

0	0	1	0
0	1	0	0
0	0	1	0
0	0	1	0
0	0	1	0
0	0	1	0
0	1	0	0
0	0	0	0
1	1	0	1
0	0	0	0
0	0	1	0
0	0	1	0
0	0	1	0
0	0	0	0
0	0	0	1
1	1	1	0
0	1	1	1
1	1	0	0
0	1	1	0
0	1	0	0
1	1	1	0
0	0	0	0
0	1	1	0
0	1	1	1
0	0	0	0
1	1	0	1
0	1	1	0
0	1	1	0
0	1	1	0
0	1	1	0
0	0	0	0
0	1	1	0
0	0	0	0
0	0	0	1
1	1	1	0
0	0	0	0
0	0	1	0
0	0	1	1
0	0	1	0
0	0	1	1
0	1	1	0
0	1	0	0
0	0	0	0
0	0	0	1
0	1	1	0
0	0	0	1
0	0	0	0
0	1	1	1
0	0	0	0
0	0	0	1
0	0	0	1
0	0	0	0
1	1	1	0

0	0	1	1
0	1	1	1
0	0	0	1
0	1	1	0
0	0	1	0
0	0	0	0
0	0	1	0
0	1	0	0
0	1	0	0
0	0	1	0
0	0	1	0
0	0	0	1
0	1	1	0
0	1	1	0
0	1	1	1
0	1	1	0
0	1	0	0
0	0	1	1
0	0	1	0
0	1	1	1
0	0	1	1
0	1	0	0
0	1	1	1
0	1	1	0
0	1	0	1
0	1	1	0
0	1	1	1
0	0	0	0
1	1	1	0
0	1	1	1
0	1	0	1
0	0	0	0
0	0	0	0
0	1	1	0
0	0	0	0
0	0	0	0
1	1	1	1
0	1	0	1
0	1	1	1
0	1	1	0
0	0	1	0
0	1	1	0
1	1	1	0
